# 3,3′-Diindolylmethane: A Promising Sensitizer of *γ*-Irradiation

**DOI:** 10.1155/2015/465105

**Published:** 2015-10-22

**Authors:** Wenjing Wang, Maomin Lv, Chaoji Huangfu, Fang Wang, Jingang Zhang

**Affiliations:** Department of Blood Biopharmaceuticals and Viral Detection, Institute of Transfusion Medicine, The Academy of Military Medical Sciences, Beijing 100850, China

## Abstract

*Purpose*. Radiotherapy is an effective treatment modality in the clinical treatment of breast cancer. The present work investigated the effect of 3,3′-diindolylmethane (DIM) on *γ*-irradiation sensitizing human breast carcinoma. *Methods*. Cell survival, intracellular ROS levels, cell cycle distribution, cell apoptosis, and expression of proteins related to apoptosis were measured with MTT assays, flow cytometry, and Western blot analysis, respectively. *Results*. *In vitro* DIM plus *γ*-irradiation arrested the activity of G2/M phase cell cycle, increased intracellular ROS level, significantly suppressed PARP (poly ADP-ribose polymerase), and enhanced *γ*-irradiation-induced apoptosis, thereby inhibiting the proliferation of MCF-7 cells. *Conclusion*. These data provide a rationale for the use of DIM as a promising sensitizer of *γ*-irradiation.

## 1. Introduction

Breast cancer (BC) is the leading cause of cancer-related female deaths. It is estimated that 1,383,500 new cases of breast cancer are diagnosed annually worldwide, 458,400 of which will die of this disease [1, 2]. Conventional radiotherapy is important in local control of breast cancer at nearly each stage [3]. Thus, an adjuvant therapy or medication in combination with radiation is more likely to increase the overall effectiveness of radiotherapy in patients with breast cancer, resulting in better survival. Despite improvement in radiotherapy efficiency, the use of radiotherapy is primarily hindered by intrinsic or acquired resistance to ionizing radiation (IR) [4, 5]. One way to enhance the radiation effect is to use radiosensitization agents.

IR kills cancer cells by inducing DNA damage and generating ROS, which in turn leads to the damage of biomolecules [6]. Besides, the accumulation of ROS can deregulates the apoptotic signaling pathway, ultimately inducing apoptosis [7–9]. Since most of radiosensitizers can upregulate ROS the ROS-mediated mechanism is considered a potential target for improving radiotherapy [10, 11].

**Figure 1:**
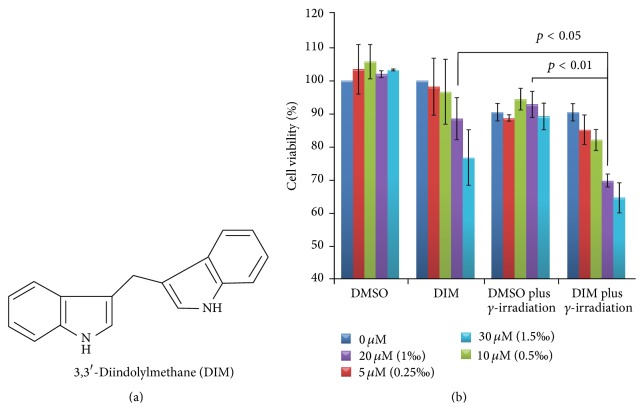
Effect of DIM on cell viability. (a) Chemical structure of 3,3′-diindolylmethane; (b) cells viability rate of MCF-7 breast cancer cells treated by DIM or/and irradiation by MTT assay *n* = 3.

Among many bioactive products derived from ingesting cruciferous vegetables 3,3′-diindolylmethane (DIM Figure 1(a)) has been investigated for preventing, inhibiting, and reversing the progression of cancer [12–14]. Several studies suggested that DIM possessed chemopreventive and therapeutic properties and was nontoxic to normal cells [15–17].

In this context, the present paper investigated the effect of DIM on the intracellular ROS level, cell cycle distribution, cell apoptosis, and expression of proteins related to apoptosis of *γ*-irradiation treated MCF-7 cells.

## 2. Materials and Methods

### 2.1. Reagents

3,3′-Diindolylmethane (purity > 98%, Figure 1(a)) solved in DMSO as storage solution was purchased from J&K SCIENTIFIC Ltd., Beijing, China. Polyclonal antibodies against LC3 (L7543) were purchased from Sigma-Aldrich, Milan, Italy, and antibodies to actin (sc-1616-R) from Santa Cruz, Biotechnology.

### 2.2. Irradiation

Cells received IR using a ^60^Co *γ*-ray source.

### 2.3. Cell Culture and Treatment

Human breast cancer cell line MCF-7, kindly provided by Professor Ming Zhao (College of Pharmaceutical Sciences, Capital Medical University, Beijing, China), was cultured in RPMI-1640 medium with 10% fetal bovine serum and 1% (v/v) penicillin-streptomycin (10000 U/mL) in 5% CO_2_ at 37°C. For all experiments, MCF-7 cells were pretreated with 20 *μ*M DIM for 2 h before exposure to *γ*-irradiation, while cells treated in parallel with respective amounts of dimethyl sulfoxide (DMSO) served as controls.

### 2.4. MTT Cell Viability Assay

MCF-7 cells, plated in triplicate wells in 96-well plates (4 × 10^3^ cells/well), were cultured overnight followed by exposure to different concentrations of DIM (5, 10, 20, and 30 *μ*M) for 48 h or 2 h before IR before being cultured for another 48 h. A medium with the same concentrations of DMSO was used as control. MTT (0.5 mg/mL) was added to the medium after incubation for 4 h. The surviving cells converted MTT into formazan, which generated a blue-purple color when dissolved in DMSO, and was measured at 570 nm using a SpectraMax M5 Microplate Reader (Molecular Devices Instruments Inc., Sunnyvale, California, USA).

### 2.5. Cell Cycle Analysis

Cells were pretreated with DIM for 2 h and then irradiated with *γ*-irradiation. After incubation for 48 h after irradiation, cells were collected, washed in PBS, and fixed in ice-cold 70% ethanol. The cells were then recentrifuged and incubated with PBS containing 200 *μ*g/mL RNase A and 5 *μ*g/mL propidium iodide (PI), and cell cycle distribution was analyzed by BD FACSCalibur Cytometry. Data was analyzed with Cellquest software (BD Biosciences, Franklin Lakes, New Jersey, USA).

### 2.6. Determination of Apoptosis

MCF-7 cells were seeded into 6-well plates at 5 × 10^5^ cells/well. After overnight incubation, they were pretreated with 20 *μ*M DIM for 2 h and then exposed to 10 Gy of *γ*-irradiation. Cells were harvested at 48 h after irradiation before being double stained with annexin V-FITC/PI using an apoptosis analysis kit (KeyGEN BioTECH, Nanjing, China) and subjected to flow cytometry analysis for detection of apoptosis. 10,000 cells per sample were analyzed by a BD FACSCalibur Cytometry (BD Biosciences, Franklin Lakes, New Jersey, USA) to quantify apoptotic cells (annexin V-FITC positive cells).

### 2.7. Measurement of ROS

Exponentially growing cells were treated with DIM (20 *μ*M) for 2 h before exposure to 10 Gy *γ*-irradiation, followed by harvesting at 2 h after irradiation. Intracellular ROS levels were measured using the Reactive Oxygen Species Assay Kit (KeyGEN BioTECH, Nanjing, China). The fluorescence intensity was proportional to the level of cellular ROS. After the indicated treatment, cells were harvested and performed according to the manufacturer's instructions. The fluorescence of the cells was monitored using flow cytometry (FACSCalibur, BD, Franklin Lakes, New Jersey, USA). ROS production was calculated as the fold increase in the fluorescence compared with that observed in the control.

### 2.8. DNA Fragmentation Detection by TUNEL Assay

For the TUNEL assay, the in situ cell-death detection kit (KeyGEN BioTECH, Nanjing, China) was performed according to previous studies [18]. In order to perform flow cytometry analysis, cells were resuspended in a final volume of 1 mL PBS. Green fluorescence (TUNEL, positive cells) was measured using a 530 nm ± 30 nm band-pass filter. A total of 10,000 events were measured on a flow cytometer (FACSCalibur, BD, Franklin Lakes, New Jersey, USA). Data was processed by CELLQUEST analysis software (Becton Dickinson).

### 2.9. Western Blot Analysis

MCF-7 cells were seeded into 6-well plates at 5 × 10^5^ cells/well. After overnight incubation, they were pretreated with 20 *μ*M DIM for 2 h and then exposed to 10 Gy of *γ*-irradiation. Cells were harvested at 48 h after irradiation, washed with ice-cold PBS, and lysed with ice-cold RIPA lysis buffer (KeyGEN BioTECH, Nanjing, China) with 1 mmol/L PMSF. Protein concentrations were calculated by BCA assay kits (Thermo Fisher SCIENTIFIC, Beijing, China). 20 *μ*g of total cellular protein was subjected to 12% SDS-PAGE and transferred to PVDF membranes (Millipore, Atlanta, Georgia, US). The membranes were blocked with 5% defatted milk powder at room temperature for 1 hr before immunoblotting was performed with primary antibodies at 4°C overnight, followed by HRP-conjugated secondary antibody at room temperature for 1 hr. Following each step, the membranes were washed five times with PBS-T for 5 min. Finally, the blots were developed using the enhanced chemiluminescence (ECL) system (Pierce Chemical, 34080).

### 2.10. Statistical Analysis

All data are presented as mean ± standard deviation (SD) of three separate experiments. Data were evaluated using SPSS for Student's *t*-test and subjected to one-way or two-way analysis of variance. Significance of the difference between the means was determined and considered significant at *p* < 0.05.

## 3. Results

### 3.1. DIM Radiosensitizes MCF-7 Cells to IR

The* in vitro* antiproliferation activity of DIM plus radiation against MCF-7 breast cancer cells was evaluated by MTT assay and the results are shown in Figure 1(b). As seen, when MCF-7 cells were exposed to DIM (0, 5, 10, 20, and 30 *μ*M) with or without *γ*-irradiation their proliferation could be suppressed to various degrees. Comparing to DMSO alone DIM concentration dependently inhibited the proliferation of the cells, but the inhibitions were significantly enhanced by *γ*-irradiation. The viability rate of the cells treated by 30, 20, and 10 *μ*M of DIM plus 10 Gy of *γ*-irradiation is 69.79% ± 1.87, 88.51% ± 6.38, and 92.85% ± 3.94, respectively.

### 3.2. DIM Increases G2/M Phase Arrest MCF-7 Cells

**Figure 2:**
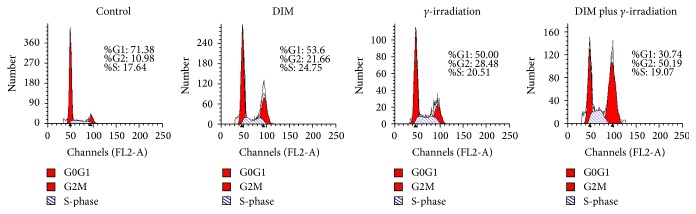
Effect of DIM on cell cycle arresting. Flow cytometric analysis of cell cycle distribution: untreated cells, DIM/radiation alone, or DIM + RT (10 Gy) were harvested for 48 h for DNA flow cytometric analysis. Percentages of G1/G0, the S phase, and G2/M phases of the cell cycle are shown.

To explore the effect of DIM plus 10 Gy of *γ*-irradiation on the MCF-7 cell cycle distribution propidium iodide, flow cytometric analyses were performed.  Figure 2 shows that through 48 h of 10 Gy of *γ*-irradiation the cell-cycle arresting in the G2/M phase is 28.48% and higher than 10.98% of the untreated cells; *γ*-irradiation-induced G2/M arrest is significantly enhanced to 50.19% by 20 *μ*M of DIM.

### 3.3. DIM Promotes *γ*-Irradiation-Induced Apoptosis in MCF-7 Cells

**Figure 3:**
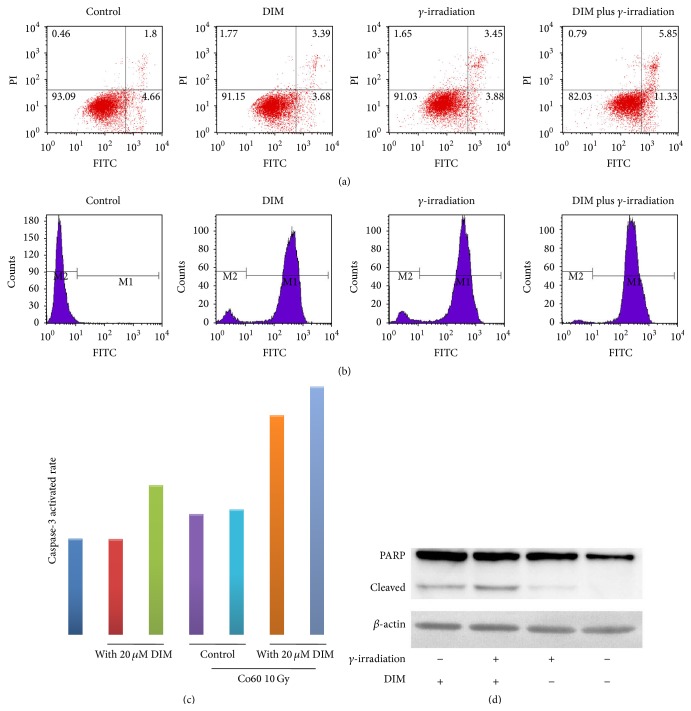
Effect of DIM on cells apoptosis. (a) Representative analyses of apoptotic MCF-7 cells by annexin V-FITC staining and flow cytometry at 48 h after they were treated with vehicle, DIM/*γ*-irradiation alone, and DIM plus *γ*-irradiation; (b) DNA fragmentation of MCF-7 breast cancer cells treated by DIM or/and *γ*-irradiation by TUNEL assay; (c) effects of DIM on the caspase-3 activity. DIM pretreated (20 *μ*M, 2 h) before exposure to *γ*-irradiation (10 Gy) can significantly increase caspase-3 activity; (d) effect of DIM (20 *μ*M) or *γ*-irradiation (10 Gy) treatment on activation of PARP cleavage. Whole cell lysates were analyzed by immunoblot analysis. Equal loading was confirmed by reprobing for *β*-actin.

To examine the effect of DIM on *γ*-irradiation-induced cell apoptosis, we evaluated the changes in the number of apoptotic cells.  Figure 3(a) shows that MCF-7 cells are resistant to *γ*-irradiation-induced apoptosis (7.3%). However, the 2 h pretreatment of 20 *μ*M of DIM increases the apoptotic cells to 17.2%.

It was hypothesized that the primary mechanism of DIM-mediated MCF-7 cells radiosensitization should be the induction of apoptosis, which was approved by DNA fragmentation, caspas-3 activity, and PARP cleavage (Figures 3(b)–3(d)). As seen, DNA fragmentation is induced in DIM and/or *γ*-irradiation group, but caspase-3 activity and PARP cleavage are enhanced only in combination group, suggesting that DIM may increase *γ*-irradiation-induced apoptosis and results in a low survival rate of cells.

### 3.4. DIM Promotes *γ*-Irradiation-Induced ROS Generation

**Figure 4:**
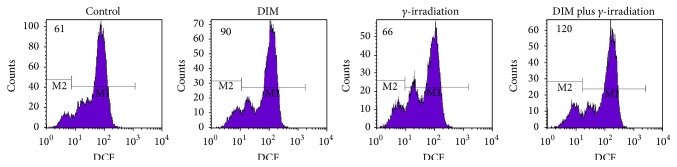
ROS generation induced by DIM. DIM (20 *μ*M) treatment for 2 h can increase intracellular ROS level in MCF-7 cells compared with untreated group; DIM pretreated (20 *μ*M, 2 h) after exposure to *γ*-irradiation (10 Gy) for 2 h can significantly increase intracellular ROS level; however, *γ*-irradiation (10 Gy, 2 h) alone presented no difference with control.

To explore the correlations between *γ*-irradiation, DIM, and ROS inhibiting cancer cells proliferation, we investigated the effects of DIM on ROS generation in MCF-7 cells (Figure 4). As seen, the ROS generated in DIM (20 *μ*M, 2 h) treated MCF-7 cells is 1.48-fold higher than that generated in the untreated MCF-7 cells, and this value is increased to 1.89-fold in DIM (20 *μ*M, 2 h) plus 10 Gy of *γ*-irradiation treated MCF-7 cells.

## 4. Discussion

Breast cancer accounts for the highest incidence of cancer and cancer-related deaths in women in both developed and developing countries. In the past 50 years, radiotherapy has played an increasing role in the treatment of breast cancer, resulted in improvements in locoregional control and survival for women receiving mastectomy and having high risk of recurrence, and allowed breast conservation in certain settings. Despite the improvement in efficiency, radiotherapy is primarily limited by intrinsic or acquired resistance to ionizing radiation. It is generally accepted that to overcome this problem radiosensitizers are needed.

DIM is considered a promising antitumor agent which could protect against tumorigenesis in multiple organs (forestomach, mammary gland, uterus, and liver) from tumorigenesis. In this study DIM plus *γ*-irradiation was shown to be concentration-dependently effective for treating breast carcinoma* in vitro*. Flow cytometry indicated a synergistic increased effect of DIM plus *γ*-irradiation on the increase of apoptotic cells.

Apoptosis is of clinical importance for radiotherapy; radiation-induced apoptosis mirrors the radiosensitivity of cancer cells [19, 20]. This study found that the measured volumes of PARP treated with 20 *μ*M of DIM alone, 10 Gy of *γ*-irradiation alone, and 20 *μ*M of DIM plus 10 Gy of *γ*-irradiation were gradually increased. These findings indicated that the induction of apoptosis was one of the primary mechanisms of DIM plus *γ*-irradiation causing death of breast cancer cells.

High level of ROS can destroy the integrity of plasma membrane, affects dynamic of actin cytoskeleton, and causes DNA damage. DCFH-DA staining and flow cytometric assays indicated that the radiosensitization of MCF-7 cells by DIM pretreatment might be as a result of the increased ROS generation. However, the mechanism of DIM generating ROS needs to be investigated in the future.

Comparing to *γ*-irradiation alone, DIM plus *γ*-irradiation can increase G2/M phase arrest, the most sensitive phase to *γ*-irradiation [21]. Our findings demonstrate that DIM is a promising candidate for a radiosensitizing agent and provide a novel strategy for improving radiotherapy against human breast cancerous tumors for the first time.
